# Comparative Evaluation of Biomedical Applications of Zinc Nanoparticles Synthesized by Using *Withania somnifera* Plant Extracts

**DOI:** 10.3390/plants11121525

**Published:** 2022-06-07

**Authors:** Bushra Hafeez Kiani, Ihsan-ul- Haq, Aiyeshah Alhodaib, Samra Basheer, Humaira Fatima, Iffat Naz, Tofeeq Ur-Rehman

**Affiliations:** 1 Department of Biological Sciences, Faculty of Basic and Applied Sciences, Female Campus, International Islamic University, Islamabad 44000, Pakistan; khansamra501@gmail.com; 2 Department of Pharmacy, Faculty of Biological Sciences, Quaid-i-Azam University, Islamabad 45320, Pakistan; ihaq@qau.edu.pk (I.-u.-H.); hfchughtai@qau.edu.pk (H.F.); tofeeq.urrehman@qau.edu.pk (T.U.-R.); 3 Department of Physics, College of Science, Qassim University, Buraydah 51452, Saudi Arabia; 4 Department of Biology, Science Unit, Deanship of Educational Services, Qassim University, Buraydah 51452, Saudi Arabia; i.majid@qu.edu.sa

**Keywords:** *Withania somnifera*, zinc oxide nanoparticles, biological activities, enzyme inhibition assays

## Abstract

Green synthesis of metal nanoparticles is of great importance in the modern health care system. In this study, zinc nanoparticles (ZnONPs) were synthesized using leaf and root extracts of *Withania somnifera* using four different solvents. ZnONPs were characterized by UV-vis spectrophotometer with a range between 350–400 nm. Scanning electron microscope revealed spherical morphology with an overall size of 70–90 nm and XRD pattern confirmed the crystalline structure. The total flavonoids, phenolic, and alkaloid contents were significantly greater in the crude extracts as compared to ZnONPs. The highest scavenging activity was observed in ZnONPs from n-hexane and ethyl-acetate extracts of roots with IC_50_ values of 27.36 µg/mL and 39.44 µg/mL, respectively. ZnONPs from methanol and aqueous extracts showed significant antibacterial activity against *Escherichia coli, Staphylococcus aureus,* and *Bacillus subtilis* while none of the extracts were found to have significant antifungal activity. Maximum cytotoxic activity was observed in ZnONPs synthesized from aqueous and n-hexane root extracts with LC_50_ values of 9.36 µg/mL and 18.84 µg/mL, respectively. The highest antidiabetic potential was exhibited by ZnONPs from n-hexane leaf extracts, i.e., 47.67 ± 0.25%. Maximum protein kinase inhibitory potential was observed in ZnONPs of ethyl-acetate extract of roots with a bald zone of 12 mm. These results indicated that *Withania somnifera*-based ZnONPs showed significant biological activities compared to crude extracts. These findings can further be utilized for in-vivo analysis of nano-directed drug delivery systems.

## 1. Introduction

Natural herbs and plants are used as conventional medicines universally. Plants have biologically active ingredients that find application in drug discovery and are used as therapeutic agents [1]. Medicinal plants have an auspicious therapeutic effect not only for cancer but also in various metabolic, cardiac, and degenerative disorders of the nervous system. They enhance memory development and prevent aging [2]. 

**Figure 1:**
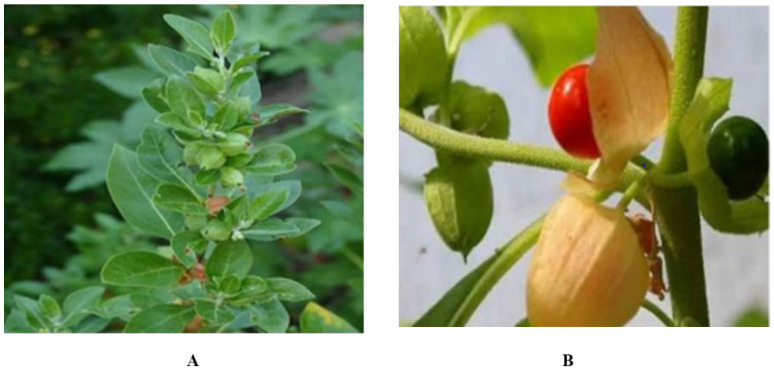
*Withania somnifera* (**A**) Plant (**B**) Fruit.

*Withania somnifera* (*W. somnifera*) is a perennial medicinal herb (Figure 1) that has anti-bacterial and anti-fungal properties. This medicinal plant falls in the Magnoliophyta division, Magnoliopsida class, *and* Solanaceae family. *W. somnifera* has been used as a medicinal shrub due to its anti-inflammatory and antioxidant features [3]. Its extract has active ingredients such as tannin, alkaloids, lactones, and flavonoids. Root, fruit, and leaf extract of *W. somnifera* have multiple applications in the pharma industry [4,5]. *Withania somnifera is* planted in East Asia, India, and Africa. It is an anti-fungal herb. Exhibits beneficial effects in tumor treatment and relaxing mind and relieves anxiety and depression [6]. It exhibits immune modulation properties and improves bone weakness, impotency, bronchitis, and muscle tension [7]. It is rich in metabolites about 62 extracted from leaves and 48 from roots [8].

Nanotechnology is an emerging field of science finds application in diagnostic and therapeutic approaches. Nanoparticles (NPs) are being used in diagnostic imaging and effective targeted drug delivery [9]. The nanoparticles are synthesized from plants, seaweeds, bacteria, fungi, and algae by the biological synthesis method. The biosynthesized nanoparticles are efficiently controlling the numerous widespread ailments with fewer side effects. Various recent studies have demonstrated that the biological synthesis of nanoparticles from plant extracts is non-hazardous rather than physical and chemical methods. Non-biological methods of NPs synthesis are costly and impose higher toxicity. Biological synthesis is quick, easy, and environment friendly. The plant extracts are effectively being used for the biological synthesis of gold, platinum, silver, cobalt, zinc oxide, palladium, magnetite, and copper nanoparticles. Many critical disorders such as human immunodeficiency virus, malaria, hepatitis, and malignant tumors are being treated by these green NPs [10].

Zinc oxide is very important in scientific research and industry as compared to other metal oxide nanoparticles, because of its exceptional features and widespread usage. It has exceptional optical, thermal, and chemical properties. Zinc Oxide nanoparticles find application in sensors, catalysis, solar energy conversion, cosmetics, fibers, paints, targeted and targeted drug-delivery due to antibacterial and luminescence features [11]. ZnONPs made from plant extracts are stable and diverse in form and magnitude as compared to those of other sources. Solvothermal synthesis, direct precipitation, reverse micelles, sol-gel method, homogeneous precipitation, sonochemical method, hydrothermal decomposition, microwave irradiation, and thermal decomposition are some of the methods used to produce these nanoparticles. The biological synthesis of nanoparticles is simple, environment friendly, and has wide antimicrobial action. The biosynthesis of ZnO nanoparticles was observed as a substitute for chemical synthesis and less toxic to the atmosphere [12]. Green synthesis is environmentally affable, economical, and quick and the product does not have contaminants. In green synthesis, there is no need for precursors and NPs of diverse shapes and magnitudes produced in bulk from plants. Usually, leaves and flowers are utilized frequently to synthesize zinc oxide nanoparticles [13]. 

Metallic nanoparticles can be synthesized by using different methods, i.e., chemical and biochemical synthesis which normally uses alkyl mercaptan, polyvinylpyrrolidone, dimethylformamide, thioanthracenol, or Tween 80 as the stabilizer and hydrazine hydrate sodium borohydride, or formaldehyde as the reducing agent to synthesize metallic nanoparticles [14]. These methods use toxic chemicals for the processing, e.g., to maintain the stability of nanoparticles and are also very expensive. This threat to the environment because of these chemical methods researchers from all over the world focused on more reliable and eco-friendly methods for the synthesis of metallic nanoparticles using some natural sources. 

Similarly, different chemical and physical methods are in use for the synthesis of ZnO NPs. These methods are very useful for the large-scale production of nanomaterials, but they have adverse effects on the environment and human health due to the use of toxic [15]. Consequently, there is an urgent need for some eco-friendly and sustainable methods for the production of metal nanoparticles [16]. Therefore, some natural sources can be used as an alternative method for the production of ZnO NPs, i.e., by using different microorganisms or different plant extracts to minimize the risk of environmental pollution [17,18]. Amid a variety of biosynthetic approaches, plant extracts are gaining global attention for the synthesis of various metal nanoparticles [19]. Polycyclic aromatic hydrocarbons (PAHs) are ubiquitous across the globe primarily due to persistent and long-term anthropogenic causes of pollution and are extremely persistent, and recalcitrant in the biosphere. PAH pollutants have been established to be highly mutagenic, toxic, carcinogenic, teratogenic, and immune-toxicogenic to various life forms. Different remediation methods involving physical, chemical, biological, and lately developed integrated approaches have been continuously applied with varying degrees of success. In this regard, recent studies have documented that ZnO NPs have shown great promise as an eco-friendly biological treatment solution for the remediation of PAHs [20].

The focus of the present research was to explore and evaluate the biological and pharmaceutical properties of ZnO nanoparticles produced from aqueous, methanol, ethyl acetate, and n-hexane obtained by the roots and leaves extracts of *Withania Somnifera.* Furthermore, the complete flavonoid and phenolic concentrations were also compared between crude extracts and ZnONPs of different solvents. Enzyme inhibition assays, i.e., protein kinase inhibition and α-Amylase inhibition assay were also carried out to analyze the efficacy of ZnONPs against these enzymes in comparison with crude extracts. 

## 2. Results 

### 2.1. Extract Recovery

To extract the phytoconstituents from *W. somnifera*, four solvents were used in a polar gradient approach. The total extract recovery was calculated for all the parts of the plant (Table 1). 

### 2.2. Synthesis of Zinc Oxide Nanaoparticles

ZnONPs were synthesized successfully by treating ethyl acetate, methanol, n-hexane, and distilled water extracts of roots and leaves with 0.01 M zinc acetate with constant stirring. After two hours of incubation, the color of the solution (pH 12) changed to off-white, which confirmed zinc nanoparticle synthesis. The solution was placed at 176 °F for oven drying overnight and the pellet was oven-dried to acquire pure nanoparticles.

### 2.3. Characterization of Zinc Nanoparticles

**Figure 2:**
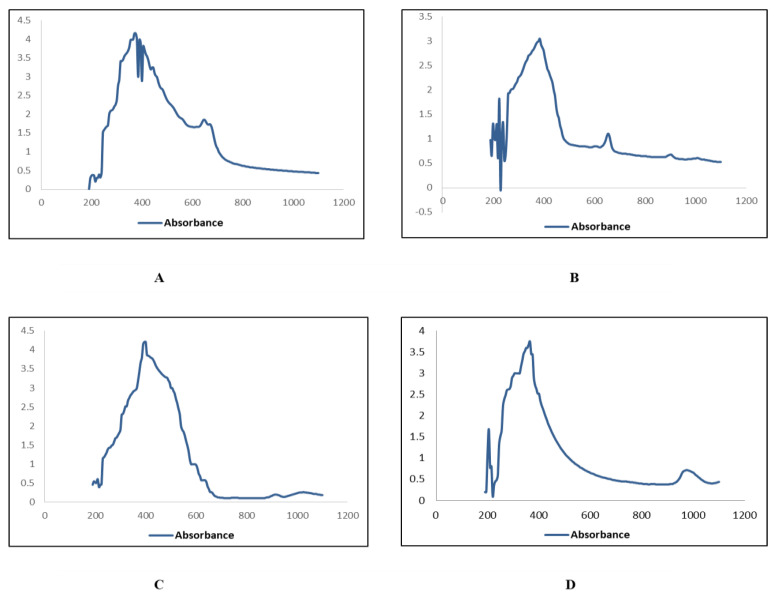
UV Visible absorption spectra of zinc oxide nanoparticles of leaves. (**A**) n_Hexane (**B**) Ethyl Acetate (**C**) Methanol (**D**) Aqueous.

**Figure 3:**
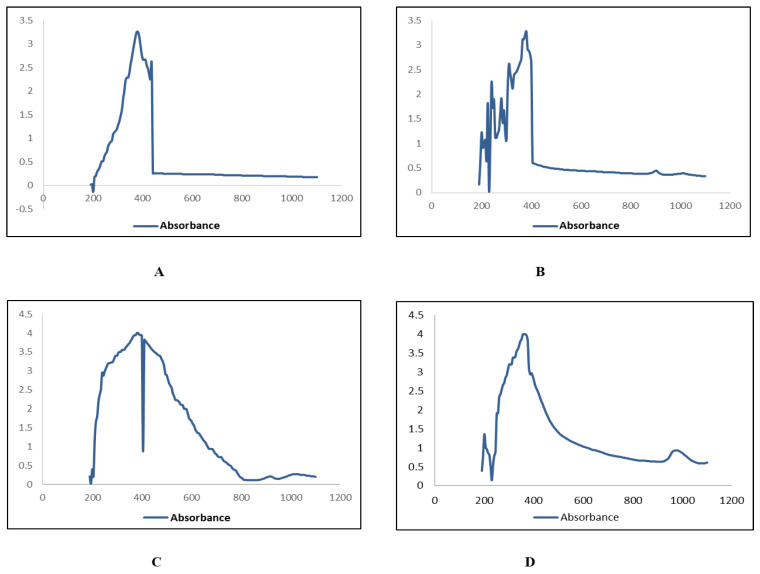
UV Visible absorption spectra of zinc oxide nanoparticles of roots; (**A**) n_Hexane (**B**) Ethyl Acetate (**C**) Methanol (**D**) Aqueous.

The UV absorbance peaks for zinc oxide nanoparticles were observed between 350 nm to 400 nm from the extracts of *W. somnifera* from leaves (Figure 2), and roots (Figure 3).

**Figure 4:**
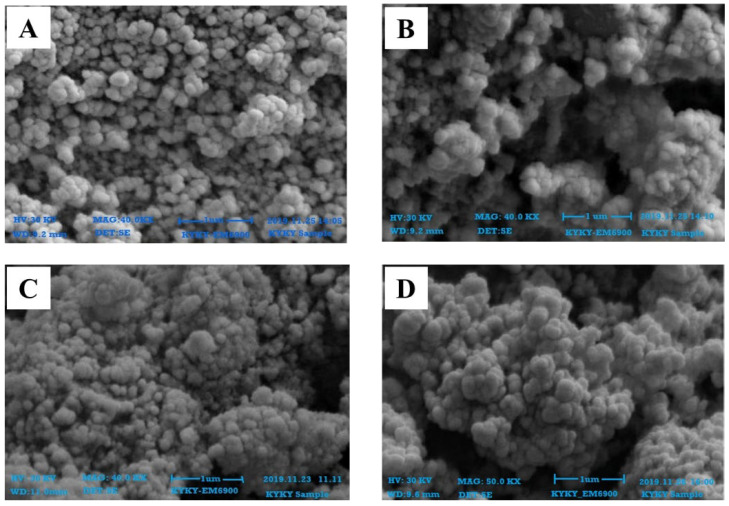
SEM images of zinc oxide nanoparticles of leaves; **(A)** n-Hexane (**B**) Ethyl Acetate (**C**) Methanol (**D**) Aqueous.

**Figure 5:**
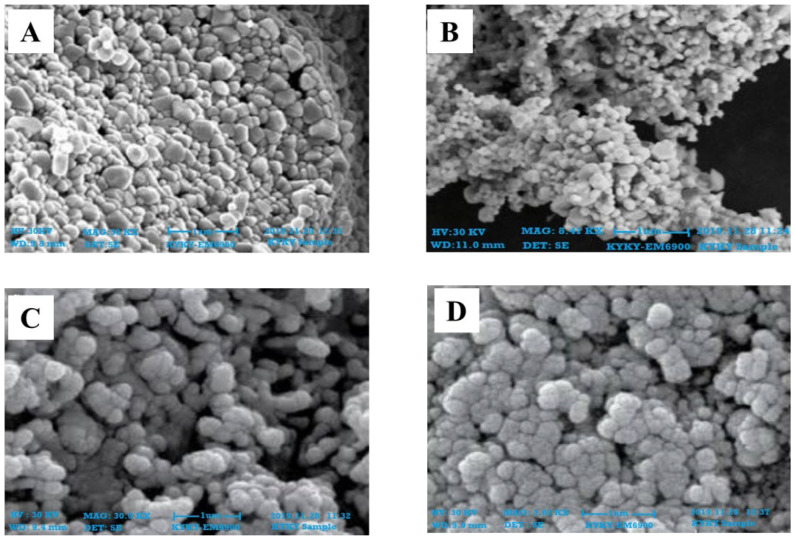
SEM images of zinc oxide nanoparticles of roots; **(A)** n_Hexane (**B**) Ethyl Acetate (**C**) Methanol (**D**) Aqueous.

The SEM results confirmed that the particles were within 100 nm for all four extracts of both leaves and roots. The size was within the range of 70–90 nm at 30 KV for the synthesized ZnONPs from leaves (Figure 4) and roots (Figure 5). The shapes were varied at some points but spherical was dominant and SEM analysis depicted the well scattered and combination of particles.

**Figure 6:**
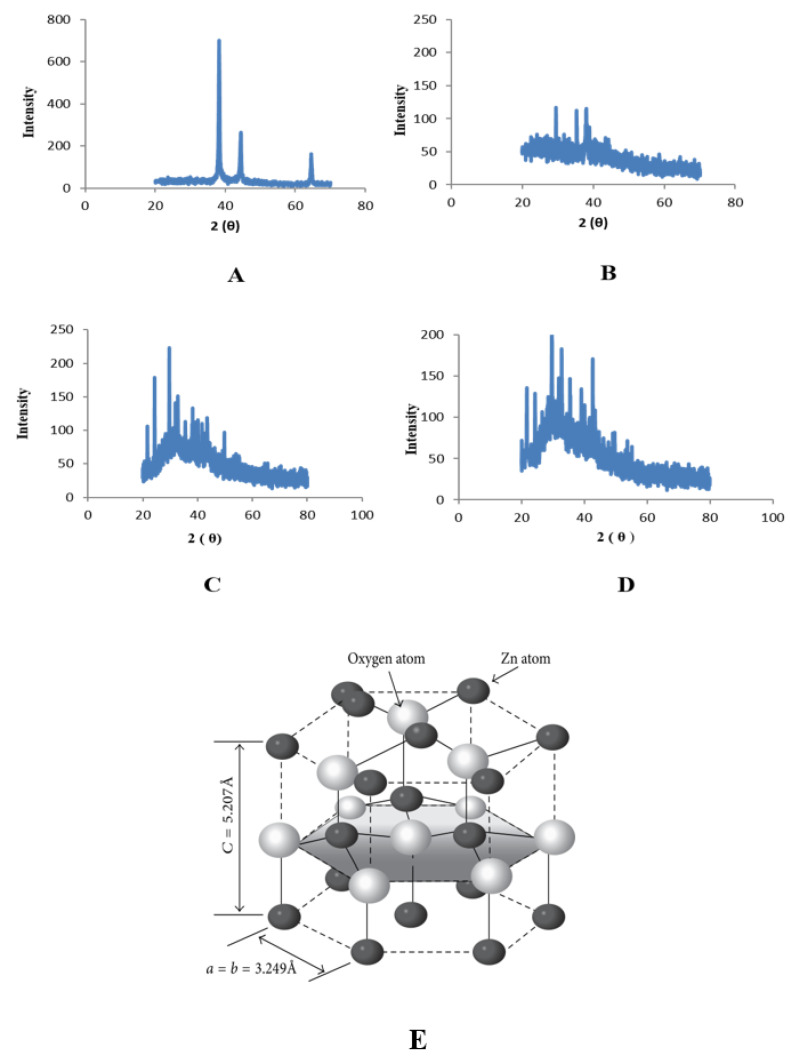
XRD peaks of zinc oxide nanoparticles of leaves; (**A**) n-Hexane (**B**) Ethyl Acetate (**C**) Methanol (**D**) Aqueous (**E**) The hexagonal wurtzite structure model of ZnO.

**Figure 7:**
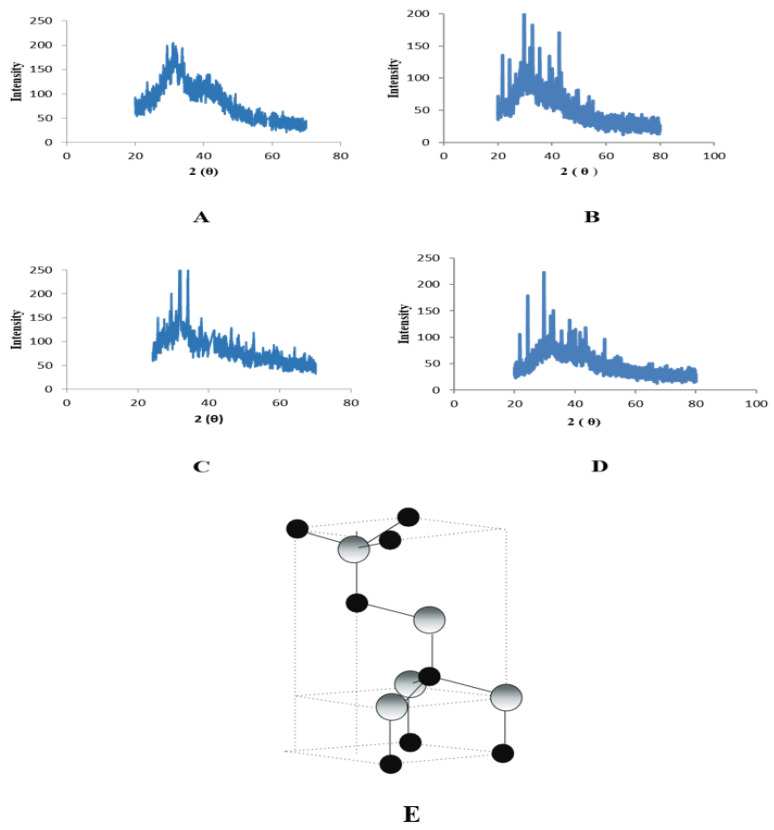
XRD peaks of zinc oxide nanoparticles of roots; (**A**) n-Hexane (**B**) Ethyl Acetate (**C**) Methanol (**D**) Aqueous (**E**) Zinc blende crystal structure of ZnO.

The presence of nanoparticles and examination of their structural properties were confirmed by an X-ray diffractometer. The spectra show the details of the crystal planes. ZnO NPs associated with leaves showed peaks with 2θ values identified at 20°, 25°, 30°, 35° and 40° which are indexed as (100), (120), (101), (220), and (311), planes (Figure 6). ZnO NPs associated with roots showed peaks with 2θ values identified at 20°, 25°, 30°, 35° and 40° which are indexed as (121), (200), (210), (220), and (311), planes (Figure 7). 

### 2.4. Phytochemical Analysis

#### 2.4.1. Total Phenolic Contents

**Figure 8:**
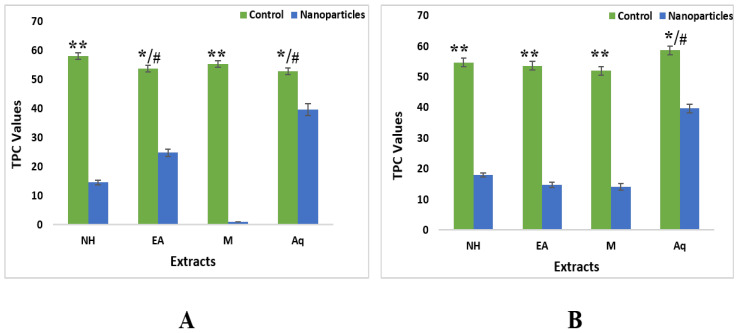
Total phenolic contents of nanoparticles and control extracts of *W. somnifera* from (**A**) Leaves (**B**) From Roots. Data are expressed as the mean ± SD. */# *p* < 0.05, ** *p* < 0.01.

The results showed that total phenolic contents have highest value in crude extracts as compared to nanoparticles. For leaves nH crude extract showed higher value (58.23 ± 0.10 GAE/g DW) as compared to ZnONPs (14.55 ± 0.18 GAE/g DW). This was followed by other three extracts, i.e., methanol [(55.32 ± 0.53 GAE/g DW—crude), (1.01 ± 0.19 GAE/g DW—ZnONPs)], Ethyl acetate [(53.92 ± 0 GAE/g DW—crude); 24.80 ± 0.37 GAE/g DW—ZnONPs)] and aqueous (53 ± 0.30 GAE/g DW—crude), 39.72 ± 0.2 GAE/g DW—ZnONPs)] (Figure 8). 

For roots, control fractions of aqueous extract showed higher values (58.66 ± 0.81 GAE/g DW) as compared to ZnONPs (39.72 ± 0.21 GAE/g DW). It was followed by nH and EA extracts, i.e., nH [(54.75 ± 0.05 GAE/g DW—crude), (18.02 ± 0.37 GAE/g DW—ZnONPs)], Ethyl acetate [(53.65 ± 0.03 GAE/g DW—crude); 14.72 ± 0.18 GAE/g DW—ZnONPs)]. The least value for nanoparticles of methanol extract showed a value of 52.05 ± 0.03 GAE/g DW in comparison with ZnONPs with a value of 14.11 ± 0.19 GAE/g DW (Figure 8).

#### 2.4.2. Total Flavonoid Contents

**Figure 9:**
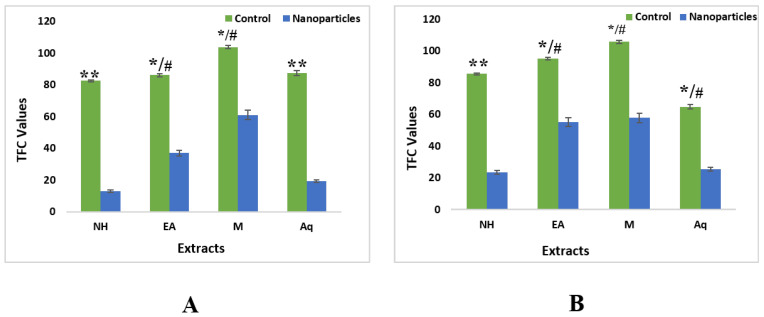
Total flavonoid contents of nanoparticles and control extracts of *W. somnifera* from (**A**) Leaves (**B**) From Roots. Data are expressed as the mean ± SD. */# *p* < 0.05, ** *p* < 0.01.

TFC was calculated as quercetin equivalents (QE). The highest TFC was observed in methanolic crude extracts of leaves, i.e., 103.65 QE/g DW ± 0.76. TFC from aqueous, EA, and nH extract were measured as 87.35 QE/g DW ± 0.35 QE/g DW, 85.98 QE/g DW ± 0.16, and 82.45 QE/g DW ± 0.09, respectively. For roots, the maximum TFC measured in methanol extract was 105.62 QE/g DW ± 0.05. Subsequently followed by EA, nH and aqueous extract at 95.23 QE/g DW ± 0.12, 85.42 QE/g DW ± 0.73 and 64.61 QE/g DW ± 0.07, respectively (Figure 9). 

Quantification of TFC of ZnONPs revealed that nanoparticles synthesized from methanolic extract of leaves had a maximum value of flavonoids, i.e., 60.92 QE/g DW ± 0.42. It was followed by EA, aqueous and nH extract, i.e., 36.91 QE/g DW ± 0, 19.24 QE/g DW ± 0.19, and 12.90 QE/g DW ± 0.05, respectively. TFC in ZnONPs synthesized from root extracts showed maximum value in methanolic extracts, i.e., 57.73 QE/g DW ± 0.42 followed by EA, aqueous and nH extract, i.e., 55.03 QE/g DW ± 0, 25.41 QE/g DW ± 0.19, and 23.51 QE/g DW ± 0.05, respectively (Figure 9).

#### 2.4.3. Total Alkaloid Contents

**Figure 10:**
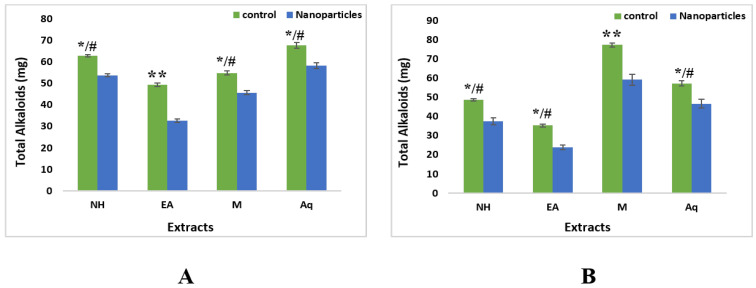
Total alkaloid contents of nanoparticles and control extracts of *W. somnifera* (**A**) From Leaves (**B**) From Roots. Data are expressed as the mean ± SD. */# *p* < 0.05, ** *p* < 0.01.

Total alkaloids results for leaves fractions of aqueous control extracts showed the highest alkaloid content (67.65 mg) and ethyl acetate showed the lowest value (54.83 mg). The same pattern was observed in nanoparticles where aqueous extracts showed the highest alkaloid content (58.24 mg) and ethyl acetate showed the lowest value (32.56 mg) as compared to n-hexane (53.76 mg) and methanol (45.65 mg) (Figure 10). 

For roots, control fractions of ethyl acetate showed the least value of 35.31 mg while in the case of nanoparticles methanolic extracts showed the highest value of alkaloid content, i.e., 59.22 mg (Figure 10).

### 2.5. Biological Activities

#### 2.5.1. Antioxidant Assay

The ZnONPs from EA and methanol extracts of leaves showed maximum scavenging activity with an IC_50_ value of 27.47 µg/mL and 28.57 µg/mL, respectively. 

**Figure 11:**
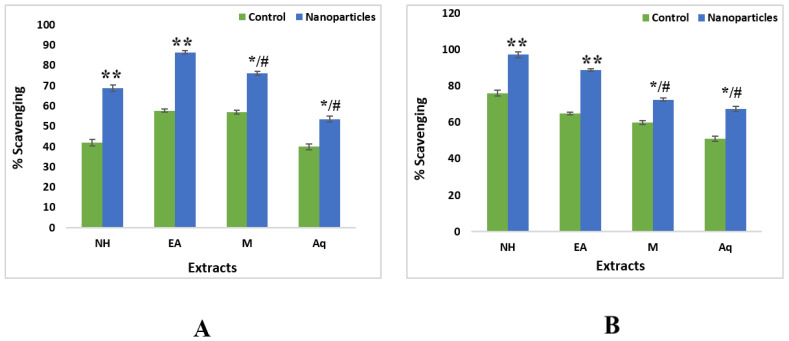
Comparative analysis of antioxidant activity of ZnONPs and crude extracts of different solvents of *W. somnifera.* (**A**) From Leaves (**B**) From Roots. Data are expressed as the mean ± SD. */# *p* < 0.05, ** *p* < 0.01.

The highest scavenging activity was observed in ZnONPs from nH and EA extract of roots with IC_50_ values of 27.36 µg/mL and 39.44 µg/mL, respectively. The crude extracts were unable to show notable free radical scavenging potential on these concentrations (Figure 11).

#### 2.5.2. Antibacterial Assay

**Figure 12:**
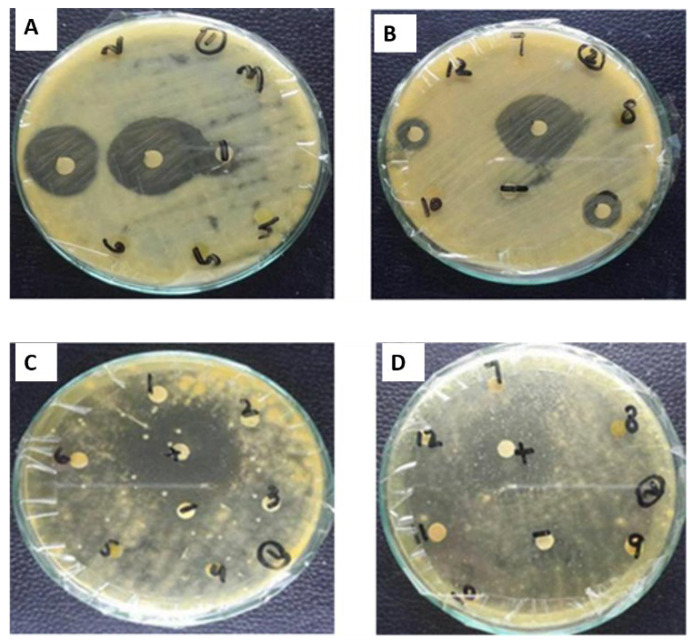
Antibacterial activity of *W. somnifera* (**A**,**B**) From Roots (**C**,**D**) From Leaves.

The antibacterial activity of the plant extracts was analyzed using the disc diffusion methodology against a variety of strains of bacteria. Test extracts that exhibited significant antibacterial activity, i.e., ≥12 mm zone of inhibition was further investigated at reduced concentrations for determination of MIC. The ZnONPs from methanol and aqueous extract of leaves showed maximum antibacterial activity against *E. coli*, i.e., 20 ± 0.76 mm (MIC100 µg/mL) and 23 ± 0.50 mm (MIC 33.3 µg/mL), respectively. The ZnONPs of leaves of aqueous extract showed activity against *B. subtilis*, i.e., 18 ± 0.5 mm (MIC 100 µg/mL) (Figure 12) (Table 2).

In the case of roots, ZnONPs from EA and aqueous extracts showed significant antibacterial activity, i.e., 21 ± 0.70 mm (MIC 33.3 µg/mL) and 18 ± 0.61 mm (MIC 100 µg/mL) against *K. pneumonia*, respectively. Aqueous extracts also showed antibacterial activity against *S. aureus* 22 ± 0.51 mm (MIC 33.3 µg/mL) and *E. coli* 20 ± 0.86 mm (MIC 33.3 µg/mL), respectively (Figure 12) (Table 2). 

#### 2.5.3. Antifungal Assay

Antifungal activity was analyzed against fungal strains by using disc diffusion methodology. None of the extracts showed significant antifungal activity. In the case of leaves, ZnONPs synthesized from methanolic extract showed mild antifungal potential against *A. Flavus*, i.e., 15 ± 0.2 mm. Slight antifungal activity against *F. solani* was also observed in the methanolic crude extract of roots and ZnONPs of methanolic extract of roots at 11 ± 0.89 mm and 14 ± 0.98 mm, respectively (Table 3).

#### 2.5.4. Cytotoxicity Activity

Cytotoxicity of the test samples was evaluated against *Artemia salina* nauplii. Overall crude extracts exhibited a significant mortality rate. ZnONPs synthesized from aqueous and nH root extract showed significant activity with LC_50_ of 9.36 µg/mL and 18.84 µg/mL, respectively. In the case of leaves, ZnONPs synthesized from EA extract exhibited eminent activity with an LC_50_ value of 20 µg/mL (Table 4). 

### 2.6. Enzyme Inhibition Assays

#### 2.6.1. Protein Kinase

Significant clear and bald zones were observed in different crude extracts and ZnONPs of extracts. Maximum bald zone of inhibition was observed in ZnONPs of EA extract of roots, i.e., 12 mm followed by nH, M, and aqueous, i.e., 10 mm, 10 mm, and 7 mm, respectively. All crude extract showed a considerable clear zone except aqueous extract which showed a bald zone 12 ± 1.00 mm.

Adequate protein kinase inhibition activity was exhibited by ZnONPs synthesized by leaves extracts. The maximum bald zone of inhibition was observed by ZnONPs of aqueous, i.e., 8 mm followed by EA, M, and nH, i.e., 8 mm, 7 mm, and 6 mm, respectively. All crude extract showed a considerable clear zone (Table 5).

#### 2.6.2. α-Amylase Inhibition Assay

α-amylase inhibition activity of crude leaf extract showed the highest activity in nH 20.68 ± 0.19% followed by methanol, EA, and aqueous, i.e., 14.88 ± 0.49%, 12.67 ± 0.98%, and 12.10 ± 0.21%, respectively. In the case of root extracts, maximum inhibition was observed by methanol 12.55 ± 0.34% followed by EA, aqueous, and nH, i.e., 10.73 ± 0.30%, 8.86 ± 0.27%, and 7.5 ± 0.25%, respectively. 

**Figure 13:**
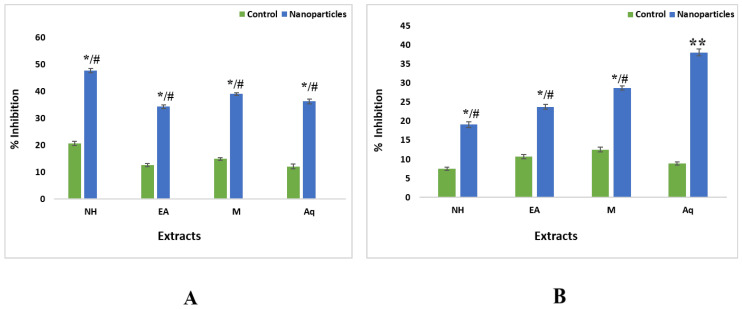
α-amylase inhibition potential of *W. somnifera* crude extracts and ZnONPs. (**A**) From Leaves (**B**) From Roots. Data are expressed as the mean ± SD. */# *p* < 0.05, ** *p* < 0.01.

ZnONPs synthesized from roots and leaves extracts demonstrated different results than crude extracts. Among ZnONPs in leaves, highest activity was detected in nH (47.67 ± 0.25%) followed by methanol, aqueous, and EA, i.e., 39.06 ± 0.17%, 36.29 ± 0.53% and 34.26 ± 0.72%, respectively. While ZnONPs from root extracts showed maximum activity in aqueous 38.01 ± 0.78% followed by methanol, EA, and nH, i.e., 28.68 ± 0.13%, 23.79 ± 0.13%, and 19.09 ± 0.28%, respectively (Figure 13). 

## 3. Discussion

Herbal products gained popularity all around the world by way of offering numerous attractive capabilities, i.e., safe, non-toxic, and easily available at a low price. Problems associated with modern-day drug treatments triggered the importance of the herbal drug industry throughout the globe [21]. 

*Withania somnifera* (L.) which is generally called ashwagandha in Sanskrit, is a perennial plant belonging to the *Solanaceae* family. The plant has been found effective in the treatment of burns, wounds, and skin problems. *W. somnifera* roots have pharmacological importance due to the presence of withanolides that are set of steroidal lactones. Its leaves are used for treating cancers and tubercular glands in Ayurvedic and Unani systems. *W. somnifera* have anxiolytic-antidepressant properties [22], antifungal [23]) antimalarial [24] apoptotic [25]. There are many studies reporting the physicochemical and pharmacological importance of *W. somnifera* [26]. 

In recent technologies, one of the rapidly developing concepts in the modern-day years is nanotechnology, which has brought high-quality development. Zinc oxide nanoparticles (ZnO NPs) are of great interest for researchers due to their potential applications such as wound healing antibacterial, antifungal, anti-inflammatory, antioxidant, anti-diabetic, and optic properties.

The current study focused on the synthesis of zinc nanoparticles using non-polar to polar extracts of *W. somnifera* as a reducing agent towards zinc acetate and additionally studied their biological activities.

Characterization of synthesized ZnONPs nanoparticles was accomplished using UV-Vis spectroscopy, SEM, and XRD. Stability and formation of ZnONPs in ethyl acetate, n-hexane, and methanol, and aqueous extracts of roots and leaves of *Withania somnifera* were confirmed by the use of UV-Vis spectroscopy, and the peak was observed at 350–400 nm (Figure 2 and Figure 3). It is reported that UV-Vis spectroscopy can be used to examine the form and length of nanoparticles [27]. SEM results reveal the regular growth of spherical nanoparticles with an average particle size of 70–90 nm (Figure 4 and Figure 5). Similar results were observed by Sulaiman et al. while working with SEM analysis of NPs of *Eucalyptus hybrida*. XRD pattern further confirmed the crystalline nature of the ZnONPs (Figure 6 and Figure 7).

Phytochemicals are very important for the treatment of several degenerative abnormalities [28,29]. Polyphenols exhibit diverse organic and pharmacological activities, e.g., Anti-inflammatory, antiulcer, antioxidant, antispasmodic, antitumor, and antidepressant activities [30]. Antioxidant properties of phenolics are attributed to the presence of ketones, hydroxyl, and methoxy functional groups [31,32].

Results of the phytochemical assays revealed the n-hexane extract contained significant phenolic content (58.23 ± 0.10 µg GAE/mg) in the case of leaves while aqueous extracts (58.66 ± 0.81 µg GAE/mg) in the case of roots. Flavonoids content was highest in methanolic extract of leaves, i.e., 103.65 ± 0.76 µg QE/mg whereas, the maximal TFC is quantified in M extract of roots, i.e., 105.62 ± 0.05 µg QE/mg. There are many reports depicting the same observations, i.e., [32] deduced total phenol content of 0.704 mg GAE/g fresh weight in water extract of *S. nigrum*. [33] used methanol extract of *C. tora* and reported a total phenol content of 180.64 ± 6.51 mg GAE/g. Uddin et al. reported a dry weight of 3.6 ± 0.089 mg GAE/g of total phenol content in n-hexane extract of *P. oleracea* [27]. A slight difference is observed in phenol content values when a comparison was carried out. This change could be the result of various plant species, carotenoids, duration, and amount of sugars, geographical variation, and ascorbic acid or extraction methods [34]. 

In the present study, NPs from EA and methanol extract of leaf showed maximum scavenging activity with IC_50_ of 27.47 µg/mL and 28.57 µg/mL, respectively. The highest scavenging activity was noted in NPs from nH and EA extract of roots with 27.36 µg/mL and 39.44 µg/mL, respectively. The crude extracts were unable to show notable free radical scavenging potential on these concentrations. Similar results were observed by [35,36,37,38,39] while working with NPs of different plant species.

Antibacterial potential of green synthesized ZnONPs from extracts of *W. somnifera* revealed maximum activity in NPs from leaves and roots extract of M and aqueous. ZnONPs from M and aqueous extract of leaves showed maximum antibacterial activity against *E. coli*, i.e., 20 ± 0.76 mm (MIC 100 µg/mL) and 23 ± 0.50 mm (MIC 33.3 µg/mL), respectively while ZnONPs of leaf aqueous extract was also active against *B. subtilis*, i.e., 18 ± 0.5 mm (MIC 100 µg/mL). This is in contrast with the statement that plant extracts from non-polar solvents exhibit strong antimicrobial potential as compared to polar extracts [40]. 

Antifungal activity of test extracts revealed that none of the crude extracts showed significant antifungal activity. While ZnONPs synthesized from M leaf extract exhibited mild antifungal potential against *A. flavus*, i.e., 15 ± 0.2 mm. Slight antifungal activity against *F. solani* was also exhibited by M crude extract of roots and ZnONPs of M extract of roots, i.e., 11 ± 0.89 mm and 14 ± 0.98 mm. This may be due to the combined effect of the antifungal potential of ZnONPs crude extract. The results suggest that ZnONPs extracts can be a good source of antimicrobial products deserving further investigation for clinical applications.

*Diabetes mellitus* is a common metabolic syndrome that is demonstrated by means of raised blood glucose levels [41]. Blood glucose level can be maintained inside tolerable variation by inhibiting the enzymes in control for hydrolyzing carbohydrates, i.e., α-amylase and α-glucosidase [42]. Inhibition of these enzymes retard starch digestion so plays a vital position in the control of diabetes. In the present study ZnONPs synthesized by roots and leaves extracts demonstrated different results than crude extracts. Among ZnONPs from leaf extracts highest activity was detected in nH 47.67 ± 0.25% while ZnONPs from root extracts exhibited maximum activity in DW 38.01 ± 0.78%.

Protein kinases are signified as oncogenic in living beings. Uncontrolled phosphorylation because of protein kinases is one of the essential factors liable for developing cancer as it alters the genetic signaling tumorigenesis [43]. Therefore, herbal merchandise with cytotoxic capability has to be investigated for the capability to arrest the kinase pastime. Protein kinases are essential for the aerial hyphae formation of Streptomyces. Test moiety able to inhibit protein kinases would have the capacity to inhibit hyphae formation main to the advent of the bald zone [44]. In the present study Maximum bald zone of inhibition in the root, part was observed in ZnONPs of EA extract of roots 12 mm while in the case of leaf extract maximum bald zone of inhibition was observed by ZnONPs of DW, i.e., 8 mm.

## 4. Materials and Methods

### 4.1. Plant Sample Collection and Identification

Fresh plants were collected from Rakhni Barkhan, Baluchistan, Pakistan. Plant was identified as *Withania somnifera* by an expert taxonomist at Quaid-i-Azam University, Islamabad, and its specimen has been preserved in the Department’s herbarium for future reference (ACC 543218). The leaves and fruit were separated and washed to clean debris and dried in shade. The dried leaves and fruits were crushed with pestle and mortar. The fine powder was stored separately for further use. 

### 4.2. Plant Extract Formulation

Extract of dehydrated *W. somnifera* leaves and fruits have been formulated through a simplified maceration process as explained by [45]. Four solvents, non-polar to the polar range, were used, i.e., n-hexane (nH), ethyl acetate (EA), methanol (MeOH), and aqueous (Aq), respectively. The 100 g of the powdered plant was soaked in 600 mL for three days of each solvent. The soaked plant material was periodically sonicated at a 25 kHz frequency. After the specified period, filtration was carried out and re-extraction was carried out with the same solvent. Using a rotary evaporator, all filtrates from the respective solvent were mixed and left to dry. After being thoroughly dried, these crudes were stored at −80 °C. 

### 4.3. Synthesis of Zinc Oxide Nanoparticles (ZnONPs)

The ZnONPs were synthesized by utilizing the methodology of [46] with few modifications. Plant extracts (50 g) were heated in a beaker up to 50–60 °C on a hot plate for 30–40 min and 5 g of zinc acetate (Sigma-Aldrich, Taufkirchen, Germany) was added directly to the heated extract with constant stirring for two hours. The color change was observed after 2 h and the extract was left to cool. The extract was transferred to Petri plates in a very thin layer. The plates were left to dry overnight in a drying oven at 60 °C. The fine and dried powder was used for the characterization process. 

### 4.4. Characterization of Zinc Oxide Nanoparticles

#### 4.4.1. UV-Visible Spectroscopy

UV-Vis spectroscopy is a widely utilized method for characterizing nanoparticles [41]. It adheres to the Beer-Lambert law [47]. The characterizing of zinc oxide nanoparticles was carried out using a wavelength of 350–400 nm. The material was examined using a spectroscope, and the spectra were monitored between 300 nm and 700 nm with a 1 nm of resolution.

#### 4.4.2. Scanning Electron Microscopy Analysis (SEM)

SEM (KYKY-EM6900 Beijing, China) examination was utilized to examine the shape and size of nanoparticles on a micrometer to nanometer level [48]. Zinc oxide nanoparticles were evaluated by placing a droplet of sample solution onto a grid evenly covered with carbon, followed by the dehydration beneath the mercury lamp for 15 min at 30 KV and photographed.

### 4.5. X-ray Diffraction Analysis (XRD)

Freeze-dried powders of the synthesized zinc oxide nanoparticles from the roots and leaves extracts of *Withania somnifera* (n-hexane, methanol ethyl acetate, and aqueous) were used for x-ray diffraction analysis. Crystalline metallic zinc nanoparticles were examined using XRD (Bruker D8 advance London, United Kingdom).

### 4.6. Phytochemical Analysis

#### 4.6.1. Total Flavonoid Concentration Assessment

The complete flavonoids concentrations were assessed as per the methodology of [49]. The extracts/samples (20 µL) were mixed with 10 µL potassium acetate, 10 µL aluminum chloride, and 160 µL of distilled water in 96 well plates. This mix was subsequently incubated for half an hour at room temperature. The absorbance was observed at a 405 nm wavelength on a microplate reader. To evaluate total flavonoid concentrations in equivalence to quercetin, the standard curve was constructed using quercetin solutions at values of 2.5–40 µg/mL. 20 µL of respective solvents were used as a negative control.

#### 4.6.2. Total Phenolic Concentration Assessment

Utilizing the Folin–Ciocalteu reagent, the complete phenolic content was measured according to [49]. At a concentration of 1 mg/µL, an amount of extract and sample solutions were made. A portion of 200 µL had to be moved to a 96-well plate, along with 90 µL of Folin–Ciocalteu reagent, which had been stirred thoroughly. Following incubating, the solution was held at room temperature for 5 min before adding 90 µL of sodium carbonate and mixing well. This resultant mixture was then put for a 60-min incubation at room temperature before being measured using a microplate reader at a 630 nm wavelength. Gallic acid (3.125–25 µg/µL) was used for plotting the standard calibration curve. Gallic acid equivalents in percentage weight by weight were used to express the total phenolic content. As a negative control, 20 µL of the respective solvents were utilized.

#### 4.6.3. Total Alkaloids Determination

The total alkaloids concentration was determined by following the methodology of [50] with some modifications. The extracts/samples were dissolved in 2N HCL and incubated at room temperature for 30 min followed by washing with 10 mL chloroform three times. pH was adjusted to neutral and then a mixture of Bromocresol green (BCG) and phosphate buffer was added in the ratio of 1:1 to the solution. The resulting mixture was extracted with chloroform, the extracts were collected, and alkaloids were quantified by using UV-Spectrophotometer (SHIMADZU UV-1800 Cambridge, United Kingdom) at 470 nm.

### 4.7. Biological Activities

The following biological activities of *W. somnifera* were performed.

#### 4.7.1. Antibacterial Assay

The disc diffusion technique was used to assess the antibacterial properties of each test extract in vitro as explained by [44]. The antibiotic effect was evaluated against 5 strains of bacteria, two of which were monoderms, namely *Staphylococcus aureus* (ATCC 6538) and *Bacillus subtilis* (ATCC 6633), and 3 of which were diderms, namely *Pseudomonas aeruginosa* (ATCC-15442), *Escherichia coli* (ATCC 15224), and *Klebsiella pneumoniae* (ATCC-1705). On nutrient agar plates, a bacterial lawn was created using a fresh culture of strains of bacteria with a seeding density of 1106 CFU/mL. Each test extract (5 µL from 20 milligrams per milliliter DMSO) was impregnated on sterile filter paper discs, with Cefaxime and roxithromycin (5 µL from 4 mg/mL DMSO) serving as positive controls and DMSO (5 µL) serving as the negative control. After 24 h of incubation at 37 °C, these discs had been put on appropriately labeled seeded agar plates, and inhibition zones surrounding every disc were determined by measuring. This test had been performed three times and the mean value was determined with the standard deviation.

The MIC was obtained using the technique described by [44]. The MIC of samples with significant inhibition zones, i.e., 12 mm, was determined using the micro broth dilution technique. Each strain’s bacterial inoculum was made using a density (5104CFU/mL) which was adjusted beforehand. In a 96-well plate, 3-fold sequential dilutions of every experiment sample were made utilizing nutrient broth up to final concentrations of 100 µg/mL, 33.33 µg/mL, 11.11 µg/mL, and 3.70 µg/mL. Bacterial cultures were rehydrated in broth culture for 11 h before being kept at 4 °C in the refrigerator.

#### 4.7.2. Antifungal Assay

The antifungal test had been performed according to the description of [44]. *Aspergillus fumigatus* (FFBP 66), *Mucor species* (FFBP 0300), *Fusarium solani* (FFBP 0291), and *Aspergillus flavis* (FFBP 0064) were all tested for antifungal activity. All fungal strains were cultured at 28 °C on 6.5% SDA (Sabouraud dextrose agar, pH 5.7) then stored in the refrigerator at 4 °C. The standard treatment was clotrimazole (4 mg/mL), while the negative control was DMSO. SDA plates holding 25 mL media were infected with 100 microliters of fungal inoculum that had been renewed. On seeded SDA plates, sterile filter paper discs containing test extracts (5 μL, 20 mg/mL DMSO), DMSO (5 microliters), and clotrimazole (5 μL, 4 mg/mL DMSO), had been inserted. These inoculated plates had been placed for 24-h incubation at 30 °C, and the inhibition zones surrounding each disc were calculated in millimeters (mm).

#### 4.7.3. Brine Shrimp Cytotoxicity Assay

In a narrow rectangle pan (22 × 32 cm) supplied with saltwater, brine shrimp (*Artemia salina*) eggs (Sera, Heidelberg, Germany) had been spawned. To form two uneven portions, a 2 mm plastic separator with several holes were placed inside the pan. The eggs (about 25 mg) had been dispersed inside the bigger section, which was shaded with aluminum foil while the other section was lighted. Phototropic napulii (brine shrimp larvae) were gathered by pipetting from the lighted side after being detached from their shells through the separator after one day of emerging.

The cytotoxicity experiment was carried out on a 96-well plate with different alphabets (A-H). 44 μL of seawater was poured in wells A and E of the microwell plate. 25 µL of seawater was poured in B, C D, F, G, H, and 6 µL of sample in A and E. 25 µL of the sample was taken from A and poured in well B, and from well B, 25 µL was added in well C. Same process was repeated for D, and 25 µL from D was discarded. This was carried out to ensure uniform dilution values. The same steps were repeated for E, F, G, and H and from H to discard. Ten shrimps were transferred into each well of the microplate and quantity was completed by adding 300 µL seawater in all wells and kept for 24 h. The survival of larvae was observed under a microscope. The test was carried out three times, and Abbott’s method was used to compute the percentages of dead larvae.

#### 4.7.4. Free Radical Scavenging Property

The 2,2,diphenyl-1-picrylhydrazyl (DPPH) test was used to determine the free radical scavenging property. The DPPH free radical test was performed using the techniques of [49]. 9.6 mg of DPPH was dissolved in 100 mL of methanol to make a solution of DPPH. The tested samples were prepared as 4 mg/mL in Dimethyl sulfoxide. Standard ascorbic acid was formed in DMSO as 1 mg/mL. In each well of a 96-well plate, an aliquot of 10 µL of test material had been introduced, accompanied by 190 µL of DPPH reagent. The mixtures had been subsequently stirred and put for incubation at 37 °C for an hour in no light. Absorbance was calculated at 515 nm by an ELISA plate reader. DMSO has been used as a negative control and ascorbic acid (AsA) had been utilized as a positive control. Every experiment had been carried out three times, with the IC_50_ values being derived using table curves software, and percentage inhibition was computed using the following equation.

% DPPH = (1 − Abs/Abc) × 100

where; “Ac” is the Absorbance of negative control and “As” is the Absorbance of the experimental sample.

### 4.8. Enzyme Inhibition Assays

#### 4.8.1. Protein Kinase Assay

In this assay, hyphae formation was observed in the purified strain of *Streptomyces* 85E according to the methodology designated by [51]. By distributing spores (mycelia fragments) from a fresh *Streptomyces* culture on sterilized plates with limited ISP4 media, a bacteria lawn was cultured. On sterilized 6-mm filter paper discs, about 5μL of every extract (20 mg/mL of dimethyl sulfoxide) was poured. The impregnated paper discs had been put directly on top of the plates inoculated with Streptomyces 85E at the peak ratio of 100 micrograms per disc. Discs injected with dimethyl sulfoxide and Surfactin were employed as negative and positive controls, correspondingly. These plates were put for a 3-day incubation at 30 °C (This is the time taken by *Streptomyces* 85E to produce hyphae), and the findings were assessed as a bald inhibition zone surrounding the samples and controls inserted discs.

#### 4.8.2. α-Amylase Inhibition Assay

The anti-diabetic capacity of sample extracts had been assessed using the standard-amylase inhibition assay with slight modifications [52]. In a 96 well plate, a reaction mix comprising of 25 µL of amylase (0.14 U/mL), 150 microliters of phosphate buffer (pH 6.8), 40 µL of starch solution (2 mg/L in potassium phosphate buffer), and 10μL sample (4 mg/mL Dimethyl sulfoxide) was put for incubation at 50 °C for half an hour before being inhibited by 20 µL of 1 molar solution of hydrochloric acid. Following this, individual wells were filled with 90 µL of iodine solution (5 mM iodine, 5 mM potassium iodide). There were no extracts of plants in the negative control, while the blank was produced with no amylase and plant extract, and both were substituted with equal amounts of the buffer. As a positive control, 250 μM acarbose was employed. After incubating, the absorbance of this reaction plate had been assessed at 540 nm. The performance had been measured in percent α-amylase inhibition per mg dry extract. It was subsequently determined using this formula:

%α − amylase inhibition = (*Os − On*)/(*Ob − On*) × 100% (1)

where *Ob* = Blank well Absorbance, *Os* = Sample Absorbance, and *On* = Negative Control Absorbance.

### 4.9. Statistical Analysis

All data are expressed as the mean ± standard deviation (SD). One-way ANOVA test is used for the determination of the *p*-value for each comparison followed by Dunnett’s test for multiple comparisons. The level of significance was established at *p* < 0.05, *p* < 0.01, or *p* < 0.001.

## 5. Conclusions

The present study presented the simple and successful method for the synthesis of zinc oxide nanoparticles by using *W. somnifera* roots and leaves extract. The resultant nanoparticles were characterized using UV-vis spectroscopy, SEM and XRD. The UV-visible spectrum showed the characteristic peaks for ZnO nanoparticles ranging from 350–400 nm. SEM analysis revealed the size from 70–90 nm. Results of the present study revealed that the phytochemical content (alkaloids, phenols, and flavonoids) of nanoparticles is higher than those of crude extracts. Moreover, the study demonstrates that ZnO nanoparticles synthesized from *W. somnifera* extract exhibit strong antibacterial, antifungal, antioxidant, antitumor, antidiabetic, and cytotoxic activities. The current study suggests that ZnONPs as well as crude extracts of *W. somnifera* prove to be a useful source of pharmaceutical outcomes and can be further explored for clinical applications. Importantly, green synthesized ZnONPs in this work are biocompatible for the tested organisms and thus may exhibit a low level of environmental hazard and toxicity. Further studies on the isolation of active compounds of this plant can be planned to discover new drugs.

## Figures and Tables

**Table 1:** Extract recovery of different parts of *W. somnifera*.

Extract Codes	Percent Extract Recovery (% *w*/*w*)	
	Roots	Leaves
nH	2.05	4.42
EA	6.54	8.87
M	12.72	14.87
Aq	17.91	19.78

nH = n-Hexane; EA = Ethyl Acetate; M = Methanol; Aq = Aqueous.

**Table 2:** Antibacterial activity and MIC values of *W. somnifera* crude extracts and ZnONPs.

Extract Codes	Antibacterial Assay									
	Diameter of Zone of Inhibition in mm (Mean ± SD) * (MIC: µg/mL)									
	S.A	MIC	B.S	MIC	P.A	MIC	K.P	MIC	E.C	MIC
Leaf										
nH	9 ± 0.28	-	3 ± 0.3	-	5 ± 0.13	-	6 ± 0.87	-	6 ± 0.51	-
EA	10 ± 0.31	-	6 ± 0.5	-	6 ± 0.31	-	7 ± 0.35	-	9 ± 0.50	-
M	9 ± 0.36	-	10 ± 0.8	100	7 ± 0.10	-	9 ± 0.76	-	13 ± 0.50	100
Aq	11 ± 0.39	-	13 ± 0.5	100	7 ± 0.10	-	12 ± 0.17	100	16 ± 0.31	100
Leaf (ZnONPs)										
nH	9 ± 0.7	-	7 ± 0.5	100	6 ± 0.31	-	7 ± 0.55	-	7 ± 0.31	-
EA	14 ± 0.3	-	9 ± 0.5	3.7	8 ± 0.15	-	11 ± 0.50	-	11 ± 0.76	3.7
M	14 ± 0.46 *	100	13 ± 0.45	33.3	9 ± 0.21	-	14 ± 0.76 *	100	20 ± 0.76 *	100
Aq	16 ± 0.31 *	100	18 ± 0.5 *	100	11 ± 0.25	-	15 ± 0.61	100	23 ± 0.50 *	33.3
Root										
nH	5 ± 0.15	-	7 ± 0.7	-	5 ± 0.15	-	7 ± 0.50	-	8 ± 0.15	-
EA	10 ± 0.17	-	8 ± 0.5	-	5 ± 0.15	-	12 ± 0.58	33.3	10 ± 0.58	-
M	14 ± 0.31	100	12 ± 0.2	100	6 ± 0.25	-	9 ± 0.31	-	13 ± 0.5	-
Aq	9 ± 0.32	-	11 ± 0.3	-	7 ± 0.33	-	13 ± 0.29	33.3	16 ± 0.50	100
Root (ZnONPs)										
nH	9 ± 0.9	-	9 ± 0.5	100	7 ± 0.31	-	7 ± 0.55	-	10 ± 0.41	-
EA	13 ± 0.12	100	9 ± 0.5	3.7	6 ± 0.55	-	21 ± 0.70 *	33.3	14 ± 0.5	3.7
M	16 ± 0.36	-	13 ± 0.31	33.3	8 ± 0.21	-	16 ± 0.96 *	100	16 ± 0.31	100
Aq	22 ± 0.51 *	33.3	12 ± 0.5	-	9 ± 0.34	-	18 ± 0.61 *	100	20 ± 0.86 *	33.3
Controls										
Rox	23 ± 0.54	1.11	17 ± 0.3	3.33	--	-	-	-	-	-
Cefix	-	-	-	-	22 ± 0.89 *	1.11	20 ± 1.2	1.11	20 ± 1.5	3.33
DMSO	-	-		-	--	-	-	-	-	-

The sample concentration was 100 µg per disc. Values (mean ± SD) are the average triplicate analysis of each plant extract (n value of 1 × 3). - = No activity. Samples showing zona e of inhibition ≥12 mm are not applicable for MIC determination. S.A = *Staphylococcus aureus*, B.S = *Bacillus subtilis*, P.A = *Pseudomonas aeruginosa*, K.P = *Klebsiella pneumoniae*, E.C = *Escherichia coli.* The values with superscript (*) letters show significantly (*p* < 0.05) different means.

**Table 3:** Antifungal activity and ZOI values of *W. somnifera* crude extracts and ZnNPs.

Extract Codes	Antifungal Assay			
	Diameter of Zone of Inhibition in mm (Mean ± SD)			
	*A. Flavus*	*A. fumigatus*	*Mucor* sp.	*F. solani*
Leaf				
nH	9 ± 0.18	-	-	-
EA	-	-	-	-
M	11 ± 0.6	-	-	-
Aq	-	-		-
Leaf (ZnONPs)				
nH	12 ± 0.4 *	-	-	-
EA	--	-	-	-
M	15 ± 0.2 *	-	-	-
Aq	-	-	-	-
Root				
nH	-	-	-	-
EA	-	-	-	-
M	-	-	-	11 ± 0.89
Aq	-	-	-	-
Root (ZnONPs)				
nH	-	-	-	-
EA	-	-	-	-
M	-	-	-	14 ± 0.98 *
Aq	-	-	-	--
Controls				
Clotrim	20 ± 0.57 *	24 ± 0.09 *	27 ± 0.57 *	28 ± 1.23 *
**DMSO**	-	-	-	-

Values (mean ± SD) are average of triplicate of each test sample (n value of 1 × 3). - = value of No activity in disc diffusion assay Clotrim = Clotrimazole. The values with superscript (*) letters show significantly (*p* < 0.05) different means.

**Table 4:** Brine shrimp lethality potential of *W. somnifera* crude extracts and ZnNPs.

Extract Codes	% Mortality (Concentration: µg/mL)				LC_50_ µg/ml
	200	100	50	25	
Leaf					
nH	70 ± 10 *	40 ± 7.5 *	2 ± 11.5 *	20 ± 0 *	130.93
EA	100 ± 0	100 ± 0	50 ± 5.7	30 ± 0	39.52
M	100 ± 0 *	40 ± 5.7 *	40 ± 0 *	30 ± 7.5 *	88.51
Aq	100 ± 0	50 ± 0	0 ± 0	0 ± 0	100
Leaf (ZnONPs)					
nH	30 ± 0	30 ± 0	0 ± 0	0 ± 0	>200
EA	100 ± 5.7	100 ± 0	70 ± 0 *	60 ± 7.5 *	20
M	30 ± 11.5	20 ± 0	10 ± 0	10 ± 5.7	>200
Aq	100 ± 0	50 ± 0	30 ± 5.7 *	20 ± 0 *	93.1
Roots					
nH	60 ± 10	30 ± 7.5	1 ± 11.5	10 ± 0	125.93 *
EA	100 ± 0	100 ± 0 *	50 ± 5.7	30 ± 0	49.22
M	100 ± 0 *	40 ± 5.7 *	40 ± 0 *	30 ± 7.5 *	78.60
Aq	100 ± 0	50 ± 0	0 ± 0	0 ± 0	100 *
Roots (ZnONPs)					
nH	100 ± 0 *	100 ± 0 *	70 ± 0 *	70 ± 0 *	18.84
EA	100 ± 7.5	90 ± 7.5	50 ± 0	40 ± 0 *	55.5
M	30 ± 5.7	16 ± 0	16 ± 0	10 ± 0	>200 *
Aq	100 ± 0	100 ± 0 *	0 ± 0	60 ± 0 *	9.36

LC_50_ of positive control (Doxorubicin) was 5.93 µg/mL. DMSO was applied as negative control and values given are expressed as mean of triplicate ± SD. The values with superscript (*) letters show significantly (*p* < 0.05) different means.

**Table 5:** Protein kinase inhibition potential of *W. somnifera* crude extracts and ZnONPs.

Plant Name	Extracts Name	Samples	Zones	Activity
*W. somnifera* Leaves	nH	N	6	Bald
		C	7	Clear
	EA	N	8	Bald
		C	7	clear
	M	N	7	Bald
		C	5	Bald
	Aq	N	8	Bald
		C	5	Clear
*W. somnifera* Roots	nH	N	10	Bald
		C	5	Clear
	EA	N	12	Bald
		C	5	Clear
	M	N	10	Bald
		C	6	Clear
	Aq	N	7	Clear/Bald
		C	7	Clear/Bald

## Data Availability

All the data is already presented in the manuscript.
